# Prediction of poststroke independent walking using machine learning: a retrospective study

**DOI:** 10.1186/s12883-024-03849-z

**Published:** 2024-09-10

**Authors:** Zhiqing Tang, Wenlong Su, Tianhao Liu, Haitao Lu, Ying Liu, Hui Li, Kaiyue Han, Md. Moneruzzaman, Junzi Long, Xingxing Liao, Xiaonian Zhang, Lei Shan, Hao Zhang

**Affiliations:** 1 School of Rehabilitation, Capital Medical University, 10 Jiaomen North Road, Fengtai District, Beijing, 100068 China; 2 Beijing Bo’ai Hospital, China Rehabilitation Research Center, Beijing, China; 3 Cheeloo College of Medicine, Shandong University, Jinan, Shandong Province China; 4 University of Health and Rehabilitation Sciences, Jinan, Shandong Province China

**Keywords:** Independent walking, Stroke, Logistic regression, eXtreme gradient boosting, Machine learning

## Abstract

**Background:**

Accurately predicting the walking independence of stroke patients is important. Our objective was to determine and compare the performance of logistic regression (LR) and three machine learning models (eXtreme Gradient Boosting (XGBoost), Support Vector Machines (SVM), and Random Forest (RF)) in predicting walking independence at discharge in stroke patients, as well as to explore the variables that predict prognosis.

**Methods:**

778 (80% for the training set and 20% for the test set) stroke patients admitted to China Rehabilitation Research Center between February 2020 and January 2023 were retrospectively included. The training set was used for training models. The test set was used to validate and compare the performance of the four models in terms of area under the curve (AUC), accuracy, sensitivity, specificity, positive predictive value (PPV), negative predictive value (NPV), and F1 score.

**Results:**

Among the three ML models, the AUC of the XGBoost model is significantly higher than that of the SVM and RF models (*P* < 0.001, *P* = 0.024, respectively). There was no significant difference in the AUCs between the XGBoost model and the LR model (0.891 vs. 0.880, *P* = 0.560). The XGBoost model demonstrated superior accuracy (87.82% vs. 86.54%), sensitivity (50.00% vs. 39.39%), PPV (73.68% vs. 73.33%), NPV (89.78% vs. 87.94%), and F1 score (59.57% vs. 51.16%), with only slightly lower specificity (96.09% vs. 96.88%). Together, the XGBoost model and the stepwise LR model identified age, FMA-LE at admission, FAC at admission, and lower limb spasticity as key factors influencing independent walking.

**Conclusion:**

Overall, the XGBoost model performed best in predicting independent walking after stroke. The XGBoost and LR models together confirm that age, admission FMA-LE, admission FAC, and lower extremity spasticity are the key factors influencing independent walking in stroke patients at hospital discharge.

**Trial registration:**

Not applicable.

## Background

Stroke is a major problem in China due to its high morbidity, mortality and disability [1]. Even with timely treatment in the acute phase, patients may still be disabled and require rehabilitation, resulting in a high economic burden [2]. A significant portion of the cost is directly attributable to the inability of stroke survivors to walk independently [3]. 40% of stroke patients who are initially unable to walk are either ambulatory or require assistance with walking three months after stroke [4]. The ability to walk independently is a key factor in a patient’s daily activities and quality of life, and regaining the ability to walk independently becomes an important goal in the rehabilitation of stroke patients with hemiplegia [5–7]. It is critical to accurately predict the subsequent recovery of walking ability in stroke patients who are unable to walk independently at the time of admission to rehabilitation [8]. In this way, clinicians and therapists can provide patients with prognosis, goal setting, treatment selection, and discharge planning, and based on accurate prediction of independent walking, the government or the patient’s family can effectively provide appropriate socioeconomic support and health care resources [9, 10].

In the field of stroke rehabilitation, studies on predictive models for walking recovery have been a hot topic [3, 11, 12]. However, some of the predictive models that have been developed are too complex to be used in a clinical setting. Therefore, there is a need to develop simple, reliable, and feasible models for predicting independent walking that can be applied to stroke patients in inpatient rehabilitation. Logistic regression (LR) has been widely used in prognostic studies of stroke patients. LR measures the relationship between a categorical dependent variable and one or more independent variables by using a probability score as the predictive value of the dependent variable [13]. LR is commonly used in predictive modeling of dichotomous outcomes in health care [14]. However, it has several drawbacks, including easy underfitting, difficulty in handling nonlinear relationships, sensitivity to outliers, and possibly poor classification accuracy [15]. Therefore, there may be limitations in applying LR to predictive modeling of prognosis in stroke patients.

As a scientific and mature modeling method, machine learning (ML) is increasingly used in epidemiological research and medicine [16, 17]. With the increasing complexity and number of data sets available, as well as multi-factor data from a variety of sources, the ML is considered to have advantages over traditional regression models [18, 19], including ease of analysis, the ability to consider a large number of variables simultaneously, and to capture complex interactions between variables.

The eXtreme Gradient Boosting (XGBoost), Support Vector Machines (SVM), and Random Forest (RF) are the more mature and widely used ML modeling algorithms. The XGBoost can be used to solve supervised learning problems using a gradient boosting framework with high accuracy, difficulty in overfitting, and scalability [20, 21]. The XGBoost has been increasingly used in healthcare research to predict or screen for prognostic factors. The SVM are one of the most popular supervised learning algorithms used for pattern recognition, classification, and regression analysis [22]. The RF is an integrated learning method that generates a collection of decision trees branching on random variables. By using the majority principle for all trees and branches, RF can make predictions with high accuracy, less overfitting and strong anti-noise ability [23]. However, the optimal model tends to vary across studies and there is a lack of models that use these ML algorithms to predict independent walking in stroke patients.

Therefore, the aim of this study was to investigate the optimal prediction of independent walking at discharge based on clinical data of stroke patients who were unable to walk independently at admission using classical logistic regression methods and three currently accepted ML models (the XGBoost, SVM, and RF model), and to explore variables related to prognosis.

## Methods

### Overview

This study protocol was approved by the medical ethics committee of China Rehabilitation Research Center (approval number 2022-141-02). Informed consent was not obtained as this was a retrospective, hospital-based study.

### Participants

Between February 2020 and January 2023, a retrospective cohort of inpatients admitted to and discharged from the neurorehabilitation unit of China Rehabilitation Research Center for first-onset stroke was studied. Patients were included if they met the following inclusion criteria: (1) were aged ≥ 18 years; (2) had a first-ever unilateral cerebral stroke; (3) were unable to walk independently at admission and had a Functional Ambulation Category (FAC) score ≤ 3. Patients were excluded according to the following criteria: (1) had other underlying neurological diseases; (2) had a diagnosis of disturbance of consciousness; (3) had unstable vital signs; (4) length of stay (LOS) < 14 days; (5) had incomplete required data.

### Data

In this study, a total of 1033 patients were screened and 778 stroke patients who met the inclusion criteria were ultimately included in the analysis. The following data were collected from 778 stroke patients (21 variables in total): age (years), sex (male or female), medical insurance (yes or no), LOS, time since onset, type of stroke (ischemic or hemorrhagic), side of stroke (left or right), lesion location (cortical, subcortical, or both), lower extremity deep vein thrombosis (yes or no), emotional disorder (yes or no), cognitive disorder (yes or no), sleep disorder (yes or no), dysphagia (yes or no), aphasia (yes or no), lower limb spasticity (yes or no), FAC score at admission, Fugl-Meyer Motor Assessment of the Lower Extremity (FMA-LE) score at admission, Fugl-Meyer Balance Assessment (FMB) score at admission, National Institutes of Health Stroke Scale (NIHSS) score at admission, Barthel Index (BI) score at admission, and FAC score at discharge. The FAC scale has been widely used to assess walking independence in stroke patients, with six levels (0–5). According to previous reports, stroke patients with fac score > 3 at discharge were defined as “independent walking”, otherwise as “non-independent walking” [24]. In this study, we used “independent walking at discharge (yes or no)” as the response variable, and the remaining 20 variables were used for prediction.

### Statistical analysis

The IBM SPSS Statistics software version 25 (IBM Corp, Armonk, USA) was used for data analysis. Categorical variables were presented as frequencies and percentages. For continuous variables, the Kolmogorov-Smirnov test was used to assess data distribution. Continuous variables were expressed as mean ± standard deviation if they fit the normal distribution; otherwise, they were expressed as medians (Q_L_, Q_U_). The χ^2^ test was used to compare categorical variables, and the Student’s t-test or the Mann-Whitney nonparametric test was used to compare continuous variables.

In this study, all enrolled patients were randomly divided into two data sets with a split ratio of 4:1. Subsequently, 80% of the patients were used for model training and 20% of the patients were used for model testing. Predictive models were constructed using the walking status at discharge (“independent walking” or “non-independent walking”) as the outcome variable. We used the “autoReg”, “XGBoost”, “e1071”, “randomForest” and “caret” packages in R software version 4.2.2 to develop and test the LR, XGBoost, SVM, and RF models. In constructing the classical LR model, we first screened the training cohort for factors associated with “independent walking” using univariate analyses. Subsequently, factors with *P* < 0.10 in the univariate analyses were included in the stepwise binary LR analysis. Due to the small number of original variables in this study and the fact that variables of lower importance may also have a beneficial effect on the training of the model, all feature variables were included in the training of the XGBoost, SVM, and RF models. The XGBoost, SVM, and RF models were optimized by either 5-fold cross-validation or hyperparameter tuning. In this study, the “pROC” package was used to plot the receiver operating characteristic (ROC) curves and calculate the area under curve (AUC) [25, 26]. The AUC was used to comprehensively evaluate the models, and the AUC of the models were compared by the Delong method [27]. The predictive performance of the models was further evaluated in terms of accuracy, sensitivity, specificity, positive predictive value (PPV), negative predictive value (NPV) and F1 score. A two-tailed *P* value < 0.05 was considered statistically significant.

## Results

### Patient characteristics

**Fig. 1:**
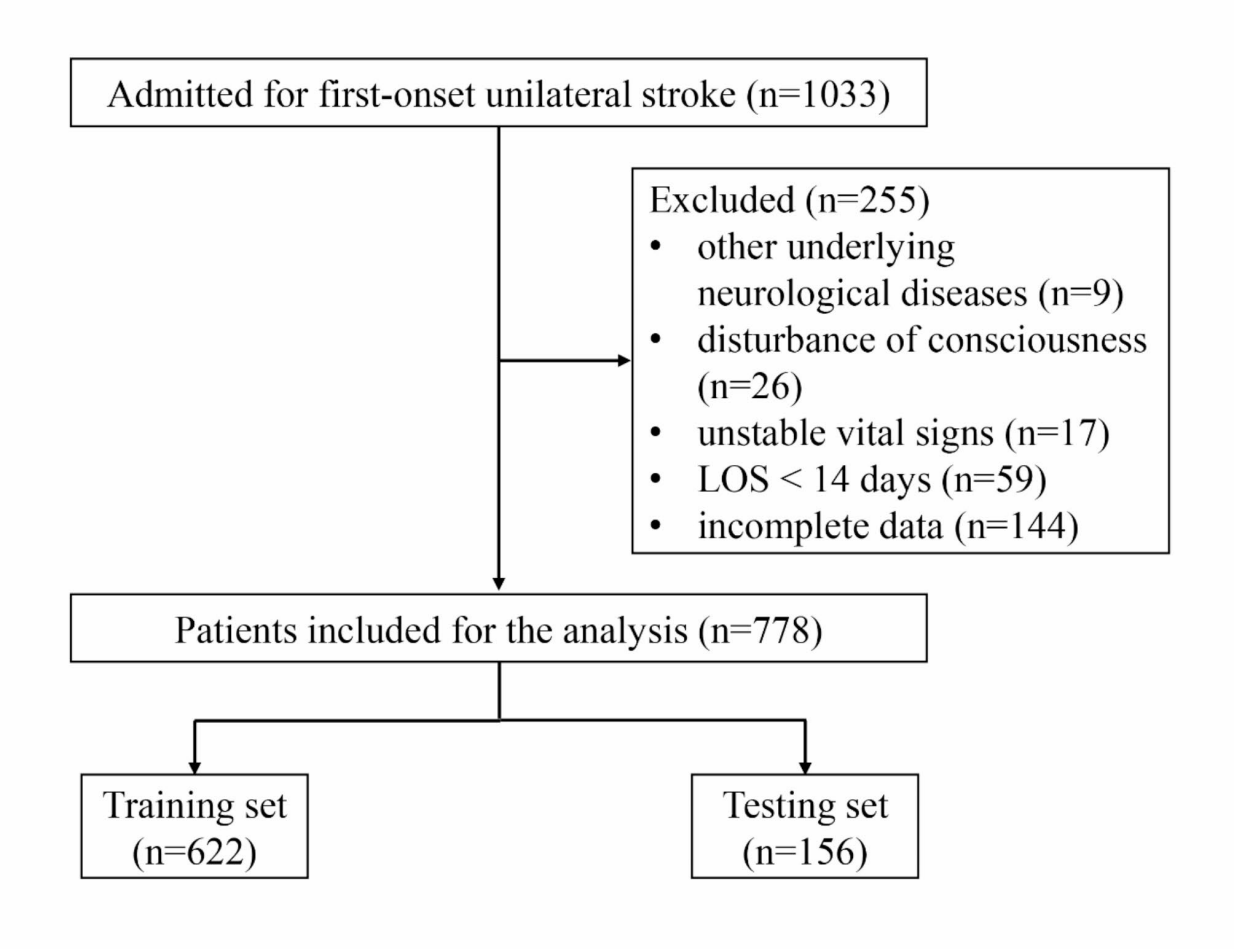
Flow-chart of participants enrolled in this study

A total of 778 stroke patients randomly assigned to the training set (*n* = 622) and the test set (*n* = 156) were finally enrolled in this study (Fig. 1). The characteristics of the training and test sets are shown in Table 1. For all the variables analyzed, there was no significant difference between the training and testing sets. Overall, 107 patients (17.20%) in the training set achieved “independent walking” at discharge and 28 patients (17.95%) in the testing set achieved “independent walking” at discharge.

**Table 1:** Comparison of the demographic and clinical characteristics of all patients, and of those in the training and testing sets

Variables	Total (*n* = 778)	Training (*n* = 622)	Testing (*n* = 156)	Statistic	*P* value
Age, years	59.00 (49.00–68.00)	59.00 (49.00–67.00)	60.50 (48.00–69.00)	Z = 0.672	0.502
Sex (male), n (%)	541 (69.54)	437 (70.26)	104 (66.67)	χ²=0.759	0.384
Medical insurance (yes), n (%)	709 (91.13)	571 (91.80)	138 (88.46)	χ²=1.721	0.190
Length of stay, n (%)				χ²=0.205	0.977
≤ 1 month	156(20.05)	125(20.10)	31(19.87)		
1–2 months	362(46.53)	291(46.78)	71(45.51)		
2–3 months	163(20.95)	130(20.90)	33(21.15)		
≥ 3 months	97(12.47)	76(12.22)	21(13.46)		
Time since onset, n (%)				χ²=0.005	0.998
≤ 1 month	65 (8.35)	52 (8.36)	13 (8.33)		
1–3 months	669 (85.99)	535 (86.01)	134 (85.90)		
≥ 3 months	44 (5.66)	35 (5.63)	9 (5.77)		
Type of stroke (ischemic), n (%)	418 (53.73)	333 (53.54)	85 (54.49)	χ²=0.045	0.831
Side of stroke (left), n (%)	427 (54.88)	345 (55.47)	82 (52.56)	χ²=0.424	0.515
Lesion location, n (%)				χ²=3.766	0.152
Cortical	112 (14.4)	86 (13.83)	26 (16.67)		
Subcortical	462 (59.38)	380 (61.09)	82 (52.56)		
Both	204 (26.22)	156 (25.08)	48 (30.77)		
Lower extremity deep vein thrombosis (yes), n (%)	162 (20.82)	132 (21.22)	30 (19.23)	χ²=0.300	0.584
Emotional disorder (yes), n (%)	348 (44.73)	278 (44.69)	70 (44.87)	χ²=0.002	0.968
Cognitive disorder (yes), n (%)	541 (69.54)	423 (68.01)	118 (75.64)	χ²=3.432	0.064
Sleep disorder (yes), n (%)	262 (33.68)	211 (33.92)	51 (32.69)	χ²=0.085	0.771
Dysphagia (yes), n (%)	190 (24.42)	157 (25.24)	33 (21.15)	χ²=1.129	0.288
Aphasia (yes), n (%)	242 (31.11)	184 (29.58)	58 (37.18)	χ²=3.359	0.067
Lower limb spasticity (yes), n (%)	195 (25.06)	162 (26.05)	33 (21.15)	χ²=1.589	0.208
FAC at admission, n (%)				χ²=0.733	0.865
0	479 (61.57)	380 (61.09)	99 (63.46)		
1	105 (13.5)	86 (13.83)	19 (12.18)		
2	90 (11.57)	74 (11.90)	16 (10.26)		
3	104 (13.37)	82 (13.18)	22 (14.10)		
FMA-LE at admission	11.00 (6.00–19.00)	11.00 (6.00–19.00)	12.00 (5.75-19.00)	Z = 0.266	0.791
FMB at admission	5.00 (2.00–8.00)	5.00 (2.00–8.00)	5.00 (1.00–8.00)	Z = 0.511	0.611
NIHSS at admission	9.00 (6.00–13.00)	9.00 (6.00–13.00)	9.50 (6.00–14.00)	Z = 0.708	0.480
BI at admission	37.50 (20.00–50.00)	40.00 (20.00–50.00)	35.00 (20.00–50.00)	Z = 0.565	0.573
Independent walking at discharge (yes), n (%)	135 (17.35)	107 (17.20)	28 (17.95)	χ²=0.048	0.826

*Note* Data are mean (standard deviation), n (%), or medians (QL, QU)

### Logistic regression model

Univariate analyses performed on the training set showed that patients who achieved “independent ambulation” at discharge were significantly different from those who did not on the variables of age, lesion location, lower extremity deep vein thrombosis, cognitive disorder, dysphagia, lower limb spasticity, FAC at admission, FMA-LE at admission, FMB at admission, NIHSS at admission and BI at admission (all *P* < 0.05) (Table 2). Subsequently, based on the results of the univariate analyses, variables with *P* < 0.10 were included in the stepwise binary LR analysis. As shown in Table 2, four variables (age, lower limb spasticity, FAC at admission, and FMA-LE at admission) were independent determinants of independent walking at discharge for stroke patients who were unable to walk independently at admission. A logistic regression model is constructed from the four influencing factors examined above, and its expression is logit (*P*) = − 2.15-0.03 × _1_ + 1.32 × _2_ + 1.61 × _3_ + 0.13 × _4_. In the formula, x_1_, x_2_, x_3_ and x_4_ represent age, no lower limb spasticity, FAC at admission = 3 and FMA-LE at admission, respectively. The Hosmer-Lemeshow goodness of fit test result on the training set was 10.211 (*P* = 0.251) with 8 degrees of freedom, and the Hosmer-Lemeshow goodness of fit test result on the test set was 6.790 (*P* = 0.560) with 8 degrees of freedom.

**Table 2:** Univariate and multivariable logistic regression model of study variables vs. independent walking at discharge in the training set

Variables	Univariate analysis			Multivariable analysis	
	Odds Ratio (95%CI)	*P* value		Odds Ratio (95%CI)	*P* value
Age	0.98 (0.97–0.99)	0.018		0.97 (0.96–0.99)	**0.013**
Sex, male vs. female	1.31 (0.82–2.11)	0.263		-	-
Medical insurance yes vs. no	0.97 (0.46–2.05)	0.930		-	-
Length of stay, vs. ≤ 1 month					
1–2 months	1.10 (0.63–1.92)	0.730		-	-
2–3 months	0.95 (0.49–1.85)	0.889		-	-
≥ 3 months	0.93 (0.43–2.01)	0.851		-	-
Time since onset, vs. ≤ 1 month					
1–3 months	0.78 (0.39–1.58)	0.497		-	-
≥ 3 months	0.35 (0.09–1.36)	0.129		-	-
Type of stroke, hemorrhagic vs. ischemic	0.92 (0.61–1.41)	0.715		-	-
Side of stroke, right vs. left	0.97 (0.64–1.48)	0.889		-	-
Lesion location, vs. cortical					
subcortical	0.48 (0.28–0.84)	0.009		-	-
both	0.45 (0.23–0.85)	0.015		-	-
Lower extremity deep vein thrombosis, no vs. yes	3.01 (1.52–5.96)	0.002		-	-
Emotional disorder, no vs. yes	1.25 (0.82–1.91)	0.303		-	-
Cognitive disorder, no vs. yes	2.60 (1.70–3.98)	< 0.001		1.60 (0.94–2.73)	0.086
Sleep disorder, no vs. yes	1.07 (0.69–1.66)	0.771		-	-
Dysphagia, no vs. yes	1.83 (1.06–3.14)	0.029		-	-
Aphasia, no vs. yes	1.38 (0.85–2.23)	0.190		-	-
Lower limb spasticity, no vs. yes	2.94 (1.60–5.42)	< 0.001		3.73 (1.80–7.72)	**< 0.001**
FAC at admission, vs. 0					
1	1.59 (0.72–3.53)	0.253		0.63 (0.25–1.54)	0.307
2	6.14 (3.26–11.57)	< 0.001		1.72 (0.78–3.77)	0.179
3	20.22 (11.16–36.63)	< 0.001		5.01 (2.40-10.46)	**< 0.001**
FMA-LE at admission	1.18 (1.14–1.21)	< 0.001		1.14 (1.10–1.18)	**< 0.001**
FMB at admission	1.40 (1.30–1.52)	< 0.001		-	-
NIHSS at admission	0.76 (0.71–0.81)	< 0.001		-	-
BI at admission	1.06 (1.05–1.08)	< 0.001		-	-

### Comparisons of logistic regression and machine learning models

**Fig. 2:**
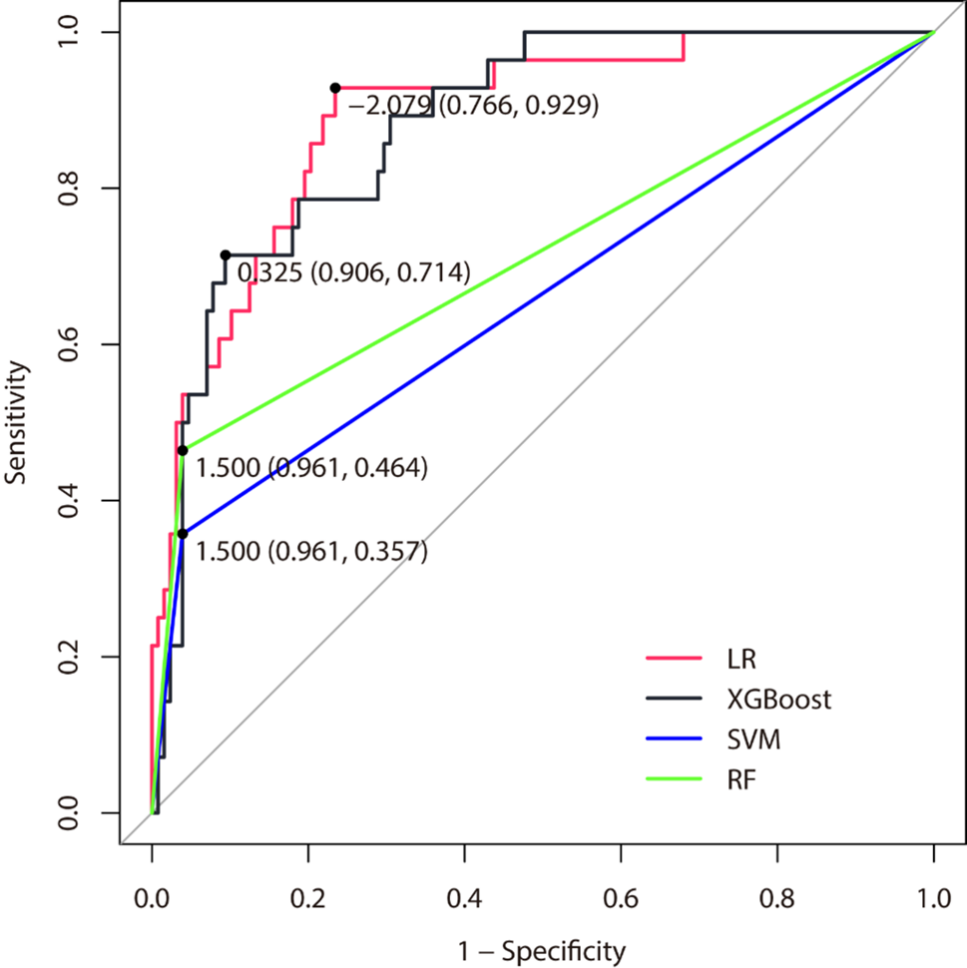
Receiver operating characteristic curve for the models

All baseline variables were used in the development of the three ML models (XGBoost, SVM, and RF) for prediction of “independent walking” at discharge. The test set was used to compare the performance of the models. In the LR model, the ROC curve was used to evaluate the discriminative ability of the prediction model, and its AUC was 0.891 (95%CI = 0.828–0.954) in the test set. The AUC of the XGBoost, SVM and RF models are 0.880 (95%CI = 0.818–0.942), 0.659 (95%CI = 0.567–0.751), and 0.713 (95%CI = 0.617–0.808), respectively. Among the three ML models, the AUC of the XGBoost model is significantly higher than that of the SVM and RF models (*P* < 0.001, *P* = 0.024, respectively). Although the LR model had a slightly higher AUC than the XGB model in the test set, there was no significant difference in the comparison (0.891 vs. 0.880, z = 0.570, *P* = 0.569). ROC curves for all models are shown in Fig. 2. Table 3 shows the number of correct predictive values of all models, based on which the accuracy, sensitivity, specificity, PPV, NPV and F1 scores of the LR, XGBoost, SVM, and RF models were calculated. These values together confirmed that the XGBoost model performed best among the three ML models, as shown in Table 4. Compared to the LR model, the XGBoost model had superior accuracy (87.82% vs. 86.54%), sensitivity (50.00% vs. 39.39%), PPV (73.68% vs. 73.33%), NPV (89.78% vs. 87.94%), and F1 score (59.57% vs. 51.16%), and the specificity was only slightly lower (96.09% vs. 96.88%).

**Table 3:** Number of correct predictive values of the LR and ML models

Model	Dataset	Observed	Predicted	
			Independent walking	Non-independent walking
LR	Training	Independent walking	40	67
		Non-independent walking	14	501
		Total	54	568
	Testing	Independent walking	11	17
		Non-independent walking	4	124
		Total	15	141
XGBoost	Training	Independent walking	77	30
		Non-independent walking	12	503
		Total	89	533
	Testing	Independent walking	14	14
		Non-independent walking	5	123
		Total	19	137
SVM	Training	Independent walking	47	60
		Non-independent walking	12	503
		Total	59	563
	Testing	Independent walking	10	18
		Non-independent walking	5	123
		Total	15	141
RF	Training	Independent walking	107	0
		Non-independent walking	0	515
		Total	107	515
	Testing	Independent walking	13	15
		Non-independent walking	5	123
		Total	18	138

**Table 4:** The performance of the LR and ML models

Model	Dataset	AUC	Accuracy	Sensitivity	Specificity	PPV	NPV	F1 score
LR	Training	0.889	86.98%	37.38%	97.28%	74.07%	88.20%	49.69%
	Testing	0.891	86.54%	39.39%	96.88%	73.33%	87.94%	51.16%
XGBoost	Training	0.972	93.25%	71.96%	97.67%	86.52%	94.37%	78.57%
	Testing	0.880	87.82%	50.00%	96.09%	73.68%	89.78%	59.57%
SVM	Training	0.708	88.42%	43.93%	97.67%	79.66%	89.34%	56.63%
	Testing	0.659	85.26%	35.71%	96.09%	66.67%	87.23%	38.53%
RF	Training	1.000	100%	100%	100%	100%	100%	100%
	Testing	0.713	87.18%	46.43%	96.09%	72.22%	89.13%	56.52%

### Predictors selection

**Fig. 3:**
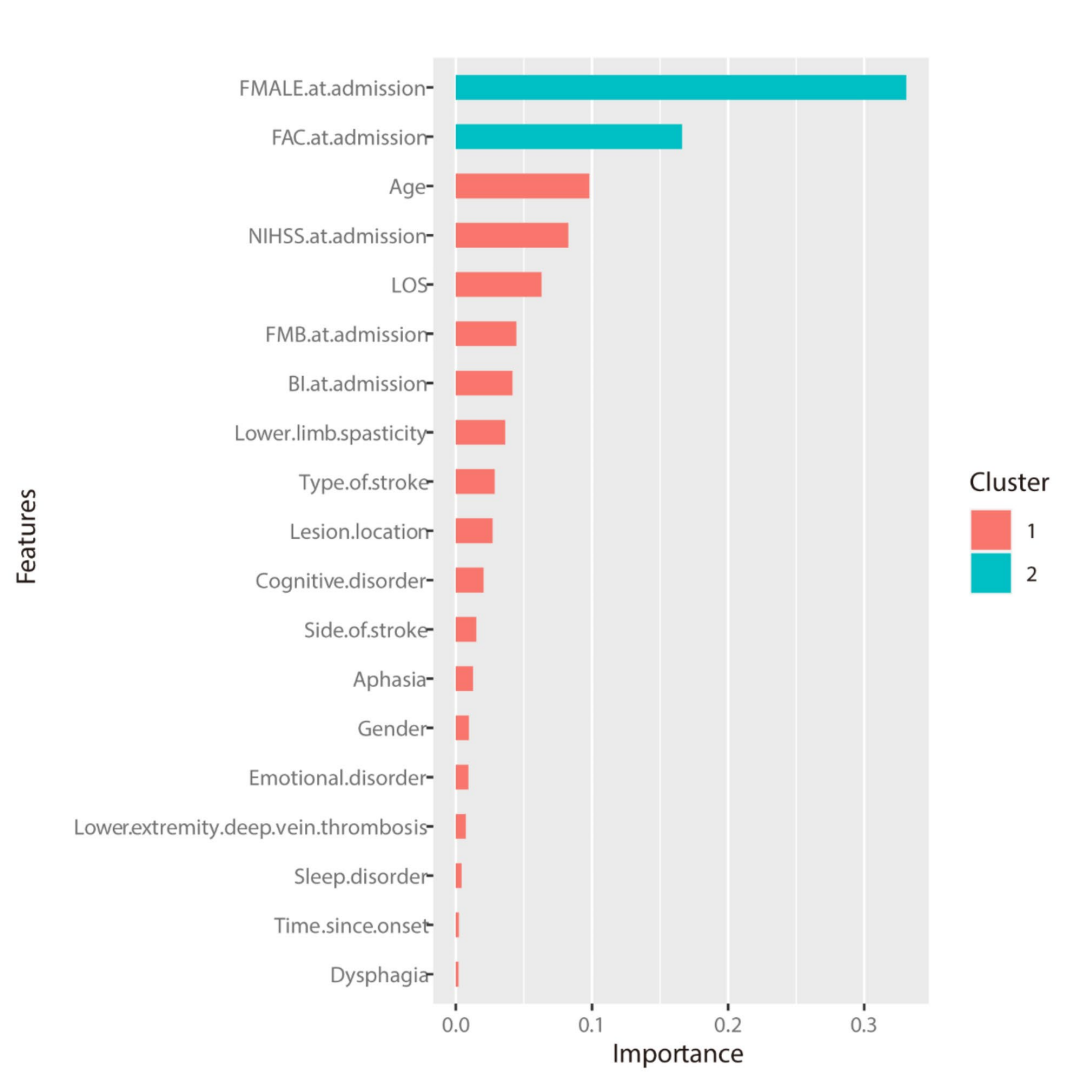
Features selected using XGBoost and the corresponding variable importance score

Stepwise logistic regression analysis showed that age, lower limb spasticity, admission FAC, and admission FMA-LE were independent predictors of independent walking in stroke patients. The XGBoost model was used to rank the importance of the feature variables, and the top ten variables are as follows: FMA-LE at admission, FAC at admission, age, NIHSS at admission, LOS, FMB at admission, BI at admission, lower limb spasticity, type of stroke, lesion location (Fig. 3).

## Discussion

It is of great importance to accurately predict the walking independence of stroke patients at the time of rehabilitation admission. In this study, we innovatively developed three machine learning algorithm-based models (XGBoost, SVM, and RF) to predict whether stroke patients would be able to walk independently at discharge from the rehabilitation center and compared them with the traditional stepwise LR model. The results show that, overall, the XGBoost model had the best predictive performance.

Most of the previous studies on related topics have used only LR analysis methods to build only one predictive model [28, 29]. However, the conventional LR analysis has its limitations, for example, it cannot well analyze the complex nonlinear relationship between variables [30]. Recently, new machine learning techniques have shown higher predictive performance compared to traditional predictive methods [31]. In this study, three commonly used machine learning algorithms (XGBoost, SVM, and RF) were selected to establish three models for predicting independent walking in stroke patients and compared with the classic LR model. First, the AUCs of the models were calculated and compared. The higher the AUC of the model, the higher the predictive value. Among the three machine learning models, the AUC of the XGBoost model was significantly higher than that of the SVM model and the RF model, suggesting that the overall performance of the XGBoost model was optimal. As a decision tree-based algorithm, XGBoost was voted the best algorithm in a machine learning and prediction competition hosted by Kaggle.com [32, 33]. Due to its best accuracy and performance, machine learning based on XGBoost algorithms has been increasingly taken seriously as a competitive alternative to regression analysis and used to predict clinical outcomes. The AUC of the two models exceeded 0.85 in both the training and test sets, indicating that the overall predictive performance of the models was good. Although the AUC of the XGBoost model was slightly lower than that of the LR model in the test set, the Delong test revealed no significant difference. Previous studies usually used multiple indicators to evaluate model performance [34, 35]. Thus, we further compared the accuracy, sensitivity, specificity, PPV, NPV, and F1 scores of the two models in the test set. Our results demonstrated that, taken together, the XGBoost model performed better than the LR model. Therefore, it was recommended that the XGBoost model be used to predict whether stroke patients who were unable to walk independently at the time of rehabilitation admission would be able to walk independently at discharge. We also suggested that future studies could consider using the XGBoost algorithm to predict other functional outcomes in stroke patients.

Step logistic regression analysis showed that age, lower extremity spasticity, FAC at admission and FMA-LE at admission were independently associated with independent walking at discharge in stroke patients. The XGBoost model ranked the importance of the variables, and the top 10 variables were FMA-LE at admission, FAC at admission, age, NIHSS at admission, LOS, FMB at admission, BI at admission, lower limb spasticity, type of stroke, lesion location. Together, the two models determined that the key variables affecting independent walking in stroke patients at discharge were age, FMA-LE at admission, FAC at admission, and lower extremity spasticity. A review of 15 studies that explored which factors predicted independent walking at 3, 6, and 12 months for in non-ambulatory people within one month of stroke, and found that younger age predicted independent walking at 3 months [3]. Similarly, we found that the younger the stroke patient, the more likely they were to walk independently at discharge. The same conclusion was also reached by Kennedy et al. [36] and Hirano et al. [12] This study also found that the presence of lower extremity spasticity prevented patients from achieving independent walking at discharge. A recent study, which found that moderate levels of plantar flexors spasticity resulted in the highest sensitivity for predicting poor gait speed performance and the highest specificity for predicting good mobility performance in post-stroke patients, supported our findings to some extent [37]. This study also showed that patients with FAC = 3 at admission were 5.01 times more likely to achieve independent walking at discharge than those who were unable to walk at all, which was consistent with the findings of Louie et al. [38]. They found that those with any ability to walk at admission (with or without therapist assistance) were 9.48 times more likely to be discharged home than those who were unable. In addition, we found that lower limb motor function was an important factor in independent walking. Hiratsuka et al. also found that lower limb motor function was an additional predictor of independent walking in a 30-day poststroke cohort [39]. Notably, the TWIST algorithm proposed by Smith et al. in 2017 incorporated trunk control test scores and hip extension strength to predict whether and when an individual patient walked independently after stroke [40]. They later built on their earlier work to examine other potential predictors, including age, knee extension strength, and Berg Balance Test score [41]. However, the trunk control test and lower limb muscle strength test were not included in the admission assessment records of patients at our hospital, and we will consider including them in future prospective studies. Some studies have also used neurophysiological or neuroimaging measures to predict walking independence in stroke patients [42–44], but one study showed that the absence of lower limb motor-evoked potentials did not preclude independent walking [45]. Although this study lacked more types of indicators to predict independent walking, we established a model with good predictive performance by using simple and easily accessible clinical data, which might be more in line with the actual clinical situation and had certain reference significance for clinical practice.

### Limitations

Undoubtedly, our study has several limitations. First, this was a retrospective, single-center study, and selection bias was inevitable. In the future, we will conduct prospective studies with larger samples to obtain more accurate results. Second, we did not have a separate data set to externally validate the predictive model established in this study, so the generalizability may not be guaranteed. Further studies using data from other hospitals are needed. Third, our prediction model used only clinical data of rehabilitation admission, whereas other studies may have incorporated imaging features, electrophysiological features, etc. In future prospective studies, we should consider using more types of data to build predictive models. Fourth, we did not follow long-term outcomes of walking function in stroke patients after discharge, and predictors of long-term outcomes in stroke patients may be different from those at discharge. Fifth, we selected only 3 commonly used machine learning algorithms to build the models and compare them, and other algorithms such as AdaBoost and neural networks deserve further investigation. However, in this study, we initially found that the XGBoost model showed better predictive performance than the LR model in predicting independent walking in stroke patients based on clinical data at the time of rehabilitation admission. Our methodology and results will inform future studies.

## Conclusions

Overall, the XGBoost model showed the best performance in predicting independent walking after stroke. The XGBoost and LR models together confirm that age, FMA-LE at admission, FAC at admission, and lower extremity spasticity are key factors affecting independent walking in stroke patients at discharge from hospital. Our study suggests that XGBoost can be used to build a predictive model of independent walking in stroke patients at discharge based on clinical data of hospitalized stroke patients, providing guidance for setting rehabilitation goals, selecting treatment plans, and making discharge plans.

## Data Availability

The datasets used and/or analysed during the current study are available from the corresponding author on reasonable request.

## Abbreviations

AUC: Area Under Curves
BI: Barthel Index
FAC: Functional Ambulation Category
FMA-LE: Fugl-Meyer Motor Assessment of the Lower Extremity
FMB: Fugl-Meyer Balance Assessment
LR: Logistic Regression
LOS: Length of Stay
ML: Machine Learning
NIHSS: National Institutes of Health Stroke Scale
NPV: Negative Predictive Value
PPV: Positive Predictive Value
RF: Random Forest
ROC: Receiver Operating Characteristic
SVM: Support Vector Machines
XGBoost: eXtreme Gradient Boosting
